# *Salmonella* illness outbreaks linked to backyard poultry purchasing during the COVID-19 pandemic: United States, 2020

**DOI:** 10.1017/S0950268821002132

**Published:** 2021-10-27

**Authors:** Megin Nichols, Lauren Gollarza, Alexandra Palacios, G. Sean Stapleton, Colin Basler, Connor Hoff, Mabel Low, Kenai McFadden, Lia Koski, Molly Leeper, Joshua Brandenburg, Beth Tolar

**Affiliations:** 1 Division of Foodborne, Waterborne and Environmental Diseases, National Center for Emerging and Zoonotic Infectious Diseases, Centers for Disease Control and Prevention, Atlanta, USA; 2 Oak Ridge Institute for Science and Education, Oak Ridge, USA; 3 CAITTA, Inc., Herndon, USA

**Keywords:** Salmonella, poultry, outbreak, COVID-19, zoonoses

## Abstract

Poultry contact is a risk factor for zoonotic transmission of non-typhoidal *Salmonella* spp. *Salmonella* illness outbreaks in the United States are identified by PulseNet, the national laboratory network for enteric disease surveillance. During 2020, PulseNet observed a 25% decline in the number of *Salmonella* clinical isolates uploaded by state and local health departments. However, 1722 outbreak-associated *Salmonella* illnesses resulting from 12 *Salmonella* serotypes were linked to contact with privately owned poultry, an increase from all previous years. This report highlights the need for continued efforts to prevent backyard poultry-associated outbreaks of *Salmonella* as ownership increases in the United States.

From March 2020 to December 2020, PulseNet, the national laboratory network for enteric disease surveillance in the United States, observed a 25% decline in the number of clinical *Salmonella* isolates uploaded with whole genome sequencing (WGS) data [1]. The PulseNet laboratory network performs WGS subtyping of isolates of *Salmonella* and other enteric pathogens such as *Listeria*, *Escherichia coli* and *Campylobacter* cultured from patient samples and analyzes sequencing data to detect enteric disease outbreaks. These surveillance activities might prompt federal, state and local public health partners to seek further information from ill people [2]. Information collected through interviews of ill people and WGS data of isolates cultured from clinical, animal and environmental samples may link human disease outbreaks to potential animal exposures and determine whether contact with animals was a source of illness. Decreased WGS data submission hinders outbreak detection and source identification.

Despite a decline in the number of clinical isolates with WGS data in 2020, outbreak-associated *Salmonella* illnesses linked to non-commercial, privately owned (from here referred to as ‘backyard’) poultry surpassed previous years' numbers and was greater than the average number of illnesses reported during the previous three years (2017–2019) (Fig. 1). Poultry are known to harbour *Salmonella* in their gastrointestinal tract and shed it in their faeces asymptomatically. Backyard poultry ownership is an established risk factor for zoonotic transmission of *Salmonella* because owners are more likely to acquire the pathogen through handling their birds or contacting environments contaminated by faeces [3, 4]. Outbreaks have been linked to backyard poultry ownership when epidemiologic data reveal a high proportion of patients that report contacting or owning poultry the week before illness onset or if laboratory testing of isolates from poultry or their environment identifies the outbreak strain of *Salmonella* [3, 4].

**Fig. 1.**
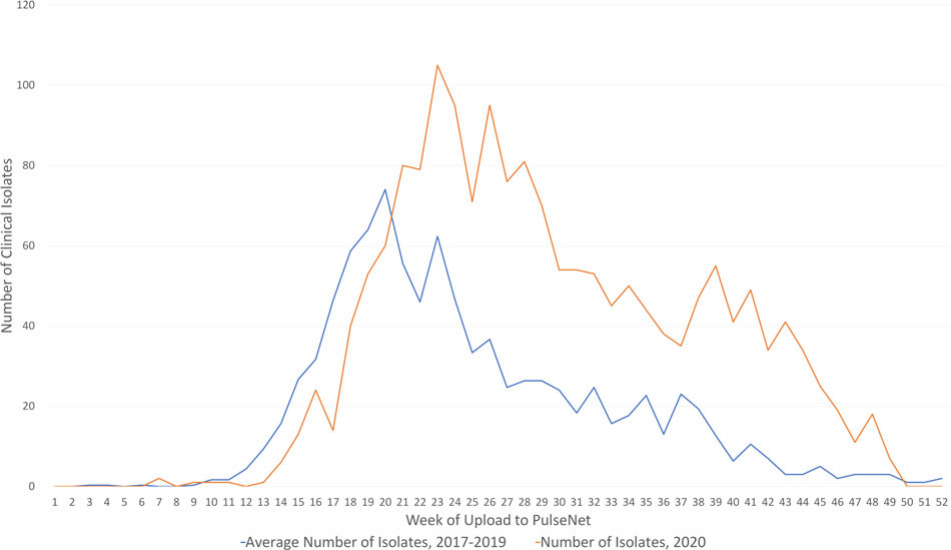
PulseNet isolate uploads: this chart represents the number of human clinical isolates linked to the backyard poultry outbreaks uploaded weekly to PulseNet in 2020 relative to a 3-year average uploaded during 2017–2019 in the United States. One clinical isolate represents one human illness. The blue line represents the average number of isolate uploads for 2017–2019. The orange line represents the number of isolate uploads in 2020.

In March 2020, an increase in human *Salmonella* Hadar infections demonstrating a high degree of genetic relatedness (<10-allele difference) was detected by PulseNet, and the Centers for Disease Control and Prevention (CDC) initiated an outbreak investigation. Additional illness outbreaks resulting from other genetically related *Salmonella* serotypes were subsequently detected by PulseNet during 2020. Cases were ultimately defined as infection with one of 12 *Salmonella* serotypes (Agona, Anatum, Braenderup, Enteritidis, Hadar, I 4,[5],12:i:-, Infantis, Mbandaka, Muenchen, Newport, Thompson and Typhimurium) demonstrating a high degree of genetic similarity based on WGS analysis within each outbreak and occurring between 1 January 2020 and 17 December 2020; these met the definition of outbreak as established by the Council of State and Territorial Epidemiologists [5]. Ill people were interviewed by state and local health departments to assess for any prevalent demographic, behavioural or epidemiological characteristics among patients infected with the outbreak strains.

Overall, 1722 outbreak-associated illnesses resulting from 12 *Salmonella* serotypes were reported from 50 states during 2020. These illnesses were grouped into 17 outbreaks based on genetic relatedness determined by WGS analysis. Patient interviews indicated a link between illness and contact with backyard poultry. This was both the highest number of backyard poultry-associated *Salmonella* outbreaks and the highest total number of outbreak-associated illnesses in a 1-year period on record for the United States (Fig. 2). Twenty-four per cent of 1722 ill people were children aged <5 years. Of 1000 people with information available, 332 (33%) were hospitalised and one death was reported. Of 879 ill people with information available, 578 (66%) reported contact with chicks and ducklings in the week before illness onset. Additionally, of 188 ill people who reported poultry contact, 72% purchased poultry for the first time. Samples collected from backyard poultry and poultry environments during the outbreaks yielded isolates nearly identical by WGS to three of the outbreak strains causing human illness (Agona, Anatum and Hadar), corroborating the link to poultry contact.

**Fig. 2.**
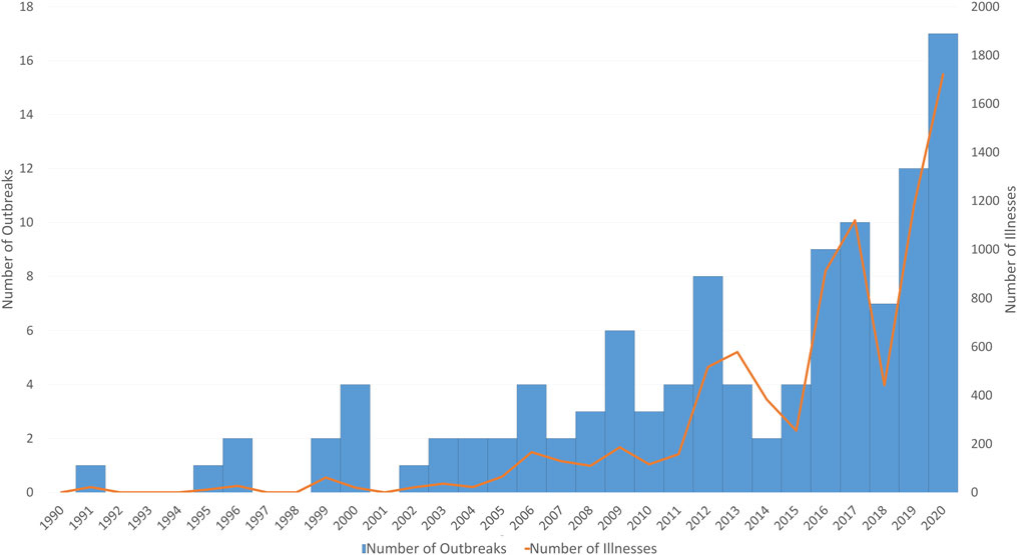
Number of outbreaks and outbreak-associated illnesses: this chart demonstrates the annual number of *Salmonella* outbreaks linked to backyard poultry (bars) and associated illnesses (orange line) reported annually during 1990 to 2020 in the United States.

This report summarises information for outbreak-associated illnesses during 2020 linked to backyard poultry. However, this is likely an underestimate of the total number of illnesses as this report does not account for sporadic human *Salmonella* infections that are not genetically related to the strains of *Salmonella* occurring as part of outbreaks. Accurately estimating illnesses, hospitalisations and deaths caused by *Salmonella* can be challenging in general because not all patients infected with *Salmonella* seek medical care, and when patients seek care, health care providers must order the appropriate diagnostic tests and ensure the case is appropriately reported to public health officials [6]. Underreporting of outbreak-associated salmonellosis during 2020 might be increased in comparison to previous years because of the impact of the COVID-19 pandemic and avoidance or delays in seeking medical care [7–9]. As a result, it is likely that more cases of *Salmonella* resulted in 2020 from contact with backyard poultry but were not detected or reported.

*Salmonella* outbreak-associated cases detected in 2020 demonstrated a similar temporal trend as previous years (Fig. 1). However, the number of outbreak-associated cases linked to backyard poultry was increased compared to 2019 when CDC identified 1134 *Salmonella* illnesses in 13 multistate outbreaks across 49 states (Fig. 2) [10]. Outbreaks of salmonellosis linked to backyard poultry demonstrate a higher likelihood of onset in the spring as a result of sale of chicks from agricultural feed stores [3]. The increase in illness during April and May in 2020 could be attributed to increases in backyard poultry sales that coincided with the COVID-19 pandemic. Agricultural stores and media outlets reported record purchasing of poultry during 2020 for egg and meat consumption and to have as pets, and a notable percentage of ill people in 2020 reported purchasing poultry for the first time [11, 12]. New poultry owners might not be aware that poultry can carry and transmit *Salmonella* through the faecal–oral route to people after handling poultry, cleaning coops, collecting eggs or not adequately washing hands. The risk of salmonellosis is higher among young children and those without previous poultry ownership experience [3, 4].

No significant changes to *Salmonella* detection, reporting or outbreak response through PulseNet or the CDC were made throughout 2020 that could explain the increase in illnesses. It is possible there is a general increase in awareness in the American population about the risks of *Salmonella* and poultry contact that prompted an increased willingness of backyard poultry owners to seek medical care, but this is not supported by available evidence to date. To further examine increases in salmonellosis linked to backyard poultry purchasing during 2020, information is needed regarding consumer purchasing behaviours, especially among first-time poultry owners and parents of young children, during the pandemic. Information about overall consumer purchasing of poultry might also help to anticipate the burden of salmonellosis during future years and inform resource allocation, health education and messaging to prevent *Salmonella* transmission from poultry to people.

## Data Availability

The data that support the findings of this study are publicly available from the Centers of Disease Control and Prevention National Outbreak Reporting System website: https://wwwn.cdc.gov/norsdashboard/. Requests for data for analysis purposes may be directed to: NORSDashboard@cdc.gov.
